# FBP1 induced by β‐elemene enhances the sensitivity of gefitinib in lung cancer

**DOI:** 10.1111/1759-7714.14750

**Published:** 2022-12-16

**Authors:** Jian Li, Ping Dai, Jing Sun, Wenyan Yu, Wei Han, Kaichun Li

**Affiliations:** ^1^ Department of Oncology Shanghai Fourth People's Hospital, Tongji University School of Medicine Shanghai China

**Keywords:** FBP1, gefitinib, lung cancer, STAT3, β‐elemene

## Abstract

**Background:**

β‐elemene is known to play a critical role in tumorigenesis as well as tyrosine kinase inhibitor (TKI) resistance in lung cancer. However, the biological function and molecular mechanism remain largely unknown.

**Methods:**

In this study, the common genes involved in gefitinib resistance and β‐elemene were identified using bioinformatic analysis. The expression of FBP1 was examined by qRT–PCR and Western blot analysis. Cell proliferation, flow cytometry, clone formation and IC50 assays were performed to assess the effects of β‐elemene and FBP1. Western blot analysis was used to evaluate apoptosis‐related gene expression. Finally, in vivo experiments were conducted to assess the crucial role of FBP1 in gefitinib‐resistant HCC827/GR cells in nude mice.

**Results:**

Screening analysis demonstrated that fructose‐1,6‐bisphosphatase (FBP1) was induced by β‐elemene and downregulated in gefitinib‐resistant lung cells. Functionally, overexpression of FBP1 inhibited proliferation and gefitinib resistance and promoted apoptosis of PC9/GR and HCC827/GR cells in vitro. Mechanistically, FBP1 impeded the nuclear translocation of p‐STAT3. The FBP1/STAT3 axis was required for FBP1‐mediated apoptosis‐related gene expression. In vivo experiments further confirmed the enhanced effects of FBP1 on lung cancer cell sensitivity to gefitinib.

**Conclusion:**

Our research indicated that β‐elemene suppressed proliferation and enhanced sensitivity to gefitinib by inducing apoptosis through the FBP1/STAT3 axis in gefitinib‐resistant lung cancer cells.

## Graphical abstract

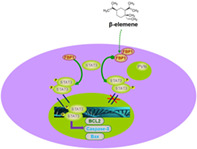

## INTRODUCTION

Lung cancer is one of the leading causes of cancer deaths worldwide.^1^ There were 828 100 newly diagnosed lung cancer cases in 2016 in China.^2^ Non‐small cell lung carcinoma (NSCLC) accounts for approximately 85% of all lung cancers with limited clinical treatment.^3^ The EGF receptor tyrosine kinase inhibitor (EGFR‐TKI) gefitinib has exhibited good initial efficacy in NSCLC patients. However, the development of acquired resistance has become a huge obstacle in clinical application.^4^, ^5^, ^6^ Therefore, further investigation of the mechanisms underlying chemoresistance has become urgent in the clinical treatment of lung cancer.

β‐elemene, a bioactive compound extracted from the herb Curcuma wenyujin, has exhibited a wide spectrum of antitumor and chemopreventive effects in a variety of tumors.^7^, ^8^ Accumulating evidence suggests that β‐elemene exerts anti‐proliferative effects on cancer cells by inhibiting cell proliferation, arresting the cell cycle, facilitating cell apoptosis, and enhancing the activity of the immune system.^7^, ^9^, ^10^, ^11^ An increasing amount of evidence has shown that β‐elemene is a clinical candidate for the cotreatment of cancer due to its safety, efficacy, and fewer adverse effects.^12^, ^13^, ^14^ β‐elemene has been reported to restore sensitivity to gefitinib by modulating the expression of p21 in NSCLC cells.^15^ Furthermore, β‐elemene combined with gefitinib exhibited a higher ability to impede lung cancer cellular proliferation, migration, and invasion in vitro.^16^ β‐elemene decreased the level of m6A methylation in gefitinib‐resistant cells via METTL3‐mediated autophagy.^17^ However, the underlying mechanisms of β‐elemene on gefitinib resistance remain largely unknown.

Fructose‐1,6‐bisphosphatase (FBP1), a key rate‐limiting enzyme in gluconeogenesis, has been shown to play an important role in several diseases, including diabetes, asthma, and cancers.^18^, ^19^, ^20^, ^21^, ^22^, ^23^ Previous research demonstrated that FBP1 expression was significantly lower in basal‐like breast cancer (BLBC) and that loss of FBP1 induced glycolysis and glucose uptake and inhibited oxygen consumption and reactive oxygen species production under hypoxia.^24^ Further investigation has shown that FBP1 acts as a metabolic tumor suppressor by inhibiting proliferation, migration, and invasion, resulting in a decrease in aerobic glycolysis and sensitizing cancer cells to chemotherapy‐induced apoptosis.

In this study, differentially expressed genes between gefitinib‐sensitive cells and gefitinib‐resistant cells were combined with bioinformatic approaches to explore the molecular mechanisms underlying β‐elemene's enhancement of gefitinib sensitivity.

## METHODS

### Bioinformatic analysis

A Bioinformatic Analysis Tool for Molecular Mechanism of Traditional Chinese Medicine (BATMAN‐TCM) database (http://bionet.ncpsb.org.cn/batman-tcm/) was used to predict potential targets of β‐elemene. The differentially expressed genes with fold change >1.5, *p* < 0.01 were analyzed using GSE123066 containing *EGFR*‐mutant HCC4006 cells and gefitinib‐resistant *EGFR*‐mutant from the Gene Expression Omnibus (GEO) database.

### Cell cultures

PC‐9 and HCC827 human lung adenocarcinoma cell lines and HEK293T human embryonic kidney cell line were purchased from the Cell Bank of the Chinese Academy of Sciences (Shanghai, China). Acquired gefitinib‐resistant PC‐9/GR and HCC827/GR cells were established by selecting parental cells with gradually elevated concentrations of gefitinib up to 2 μM for 2 months as described previously. Cells were maintained in RPMI 1640 medium (Hyclone) supplemented with 10% fetal bovine serum (Invitrogen) and 1% penicillin–streptomycin (Invitrogen) at 37°C in a humidified incubator containing 5% CO_2_.

The β‐elemene injection (National Medical Product Administration Approval Number: Chinese medicine H10960114) was obtained from Dalian Huali Jingang Pharmaceutical (Dalian, China). The STAT3‐specific activator colivelin (sc‐361 153) was purchased from Santa Cruz Biotechnology (TX, USA).

### Reverse transcription‐quantitative polymerase chain reaction (RT–qPCR)

Cells from each group were harvested, and total RNA was extracted from cells using TRIzol Reagent (Invitrogen) according to the manufacturer's protocol. A PrimeScript RT Master Mix kit (Takara, Japan) was used for the generation of cDNA. The SYBR Green Premix Pro Taq HS qPCR Kit (Accurate Biology) was used to perform RT–qPCR. The 2 − ΔΔCt method was used to calculate Gli1 mRNA expression. The primers were synthesized by Sangon (Shanghai, China), and the primers (5′‐ > 3′) used for qRT–PCR were as follows: FBP1 forward, GAAGCCTCTCATCTTATGGCATT; FBP1 reverse, CCTCATCACCTGACTCCACAA; GAPDH forward, GAGTCCACTGGCGTCTTCAC; and GAPDH reverse, ATCTTGAGGCTGTTGTCATACTTCT.

### Construction of plasmids and transfection experiments

The lentivirus‐mediated FBP1 overexpression was constructed by Gene Pharma Biotechnology Co., Ltd. pLKO.1 ‐ TRC cloning vector‐encoded shRNA targeting FBP1 was constructed as previously described.^25^ Lentiviruses were packaged using a Lentiviral Packaging kit (Gene Pharma). The oligos were as follows: forward sequence: 5′‐ CCGGCCCAGATAATTCAGCTCCTTACTCGAGTAAGGAGCTGAATTATCTGGGTTTTTG‐3′; reverse sequence: 5′‐ AATTCAAAAACCCAGATAATTCAGCTCCTTACTCGAGTAAGGAGCTGAATTATCTGGG‐3′. Successful insertion was verified by sequencing. Stable overexpression or knockdown cells were established by further screening with puromycin (Sigma–Aldrich).

### Cell viability assay

Cell counting kit‐8 (CCK‐8, Dojindo) assays were used to evaluate cell proliferation and drug sensitivity. For cell proliferation, 3 × 10^3^ cells were seeded into each well of a 96‐well plate. Ten microliters of CCK‐8 solution was added to each well at 24, 48, 72, and 96 h. Absorbance was measured by a Tecan microplate reader at a wavelength of 450 nm after incubation for 1 h at 37°C. For gefitinib sensitivity, after different treatments, cells were treated for 24 h with gefitinib (0, 2, 4, 8, 16, 32, 64, 128, or 256 μM). Then, 10 μl of CCK‐8 solution was added to each well, and the absorbance was measured. The half maximal inhibitory concentration (IC50) was calculated based on the cell survival ratio using GraphPad Prism.

### Colony formation assay

PC‐9/GR and HCC827/GR cells were seeded in 6‐well dishes (800/well). After different treatments, the medium was changed every 2 days. After incubation at 37°C for 14 days, the cells were fixed using 4% paraformaldehyde and stained for 30 min with 0.5% crystal violet. Images were captured using a digital camera (Nikon), and the visible colonies were counted manually under a light microscope (Leica, Germany).

### Flow cytometry

Cells were harvested and washed with precooled PBS. Then, the cells were stained with 5 μl of annexin V‐FITC (annexin V‐FITC/propidium iodide (PI) apoptosis detection kit I, BD Biosciences) according to the manufacturer's instructions.

### Western blot analysis

Protein from different groups of cells was extracted using cell lysis buffer (Beyotime Biotechnology) supplemented with phosphatase inhibitor. Then, the protein concentration was measured by the bicinchoninic acid (BCA) method (ThermoFisher). The following steps were performed as a standard protocol. Primary antibodies were used as follows: FBP1 antibody (no 52804, Cell Signaling Technology), Bcl‐2 (D55G8) rabbit mAb (no 4223, Cell Signaling Technology), cleaved caspase‐3 (Asp175) antibody (no. 9661, Cell Signaling Technology), Bax antibody (no. 2772, Cell Signaling Technology), STAT3 polyclonal antibody (no. 12640, Cell Signaling Technology), phospho‐Stat3 (Tyr705) (no. 9145, Cell Signaling Technology), histone H3 antibody (no. 9715, Cell Signaling Technology), GAPDH mouse monoclonal antibody (CW0100, CWbio), and anti‐β‐tubulin mouse monoclonal antibody (CW0098, CWbio).

### Murine xenograft model

Five‐week‐old BALB/c male nude mice were purchased from Shanghai ZY Department of Laboratory Animal (Shanghai, China). All animal operations and procedures were authorized by the Animal Care Committee of Shanghai ZY Department of Laboratory Animals. Nude mice were randomly divided into two groups: HCC827/GR cells transduced with either vector or FBP1 in serum‐free DMEM were subcutaneously injected into the flanks of mice, and tumor volumes were measured every 5 days. Finally, the mice were euthanized with 5% pentobarbital and sacrificed 28 days after subcutaneous injection. Then, tumors were harvested, photographed, and weighed.

### Statistical analysis

Data are presented as the mean ± standard deviation (SD). The two‐sided Student's *t*‐test was used for comparisons of two independent groups. One‐way analysis of variance was used for comparisons of more than two groups. *p* < 0.05 was considered statistically significant.

## RESULTS

### FBP1 was induced by β‐elemene in gefitinib‐resistant PC9/R and HCC827/GR cells

**FIGURE 1:**
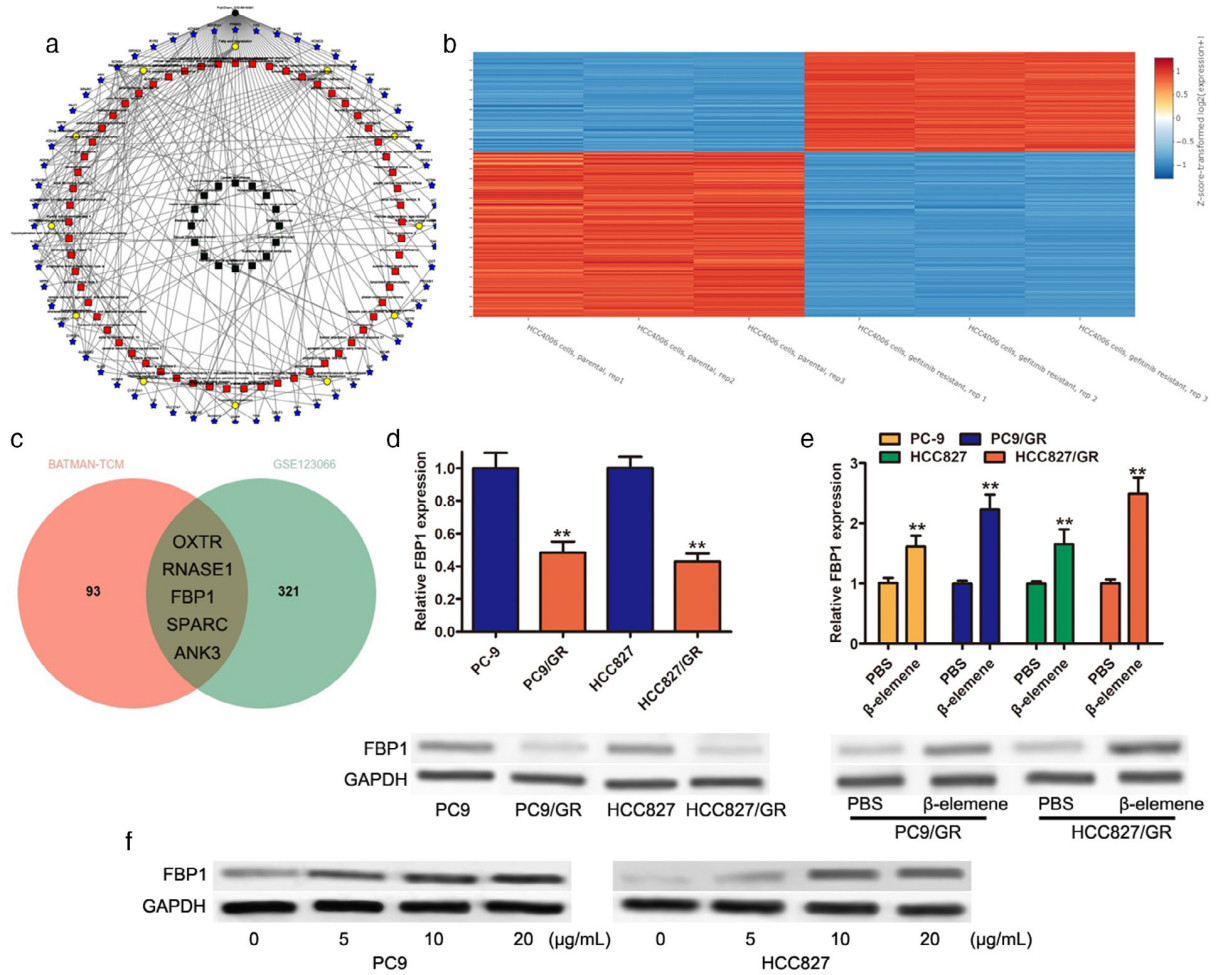
Downregulation of FBP1 in gefitinib‐resistant lung cells. (a) Simplified β‐elemene‐key target network. (b) Heatmap depicting the differentially expressed genes between parental HCC4006 cells and gefitinib‐resistant HCC4006 cells based on GSE123066 datasheets. (c) Venn diagram of β‐elemene targets and the downregulated genes in gefitinib‐resistant HCC4006 cells from GSE123066 datasheets. (d) The mRNA and protein levels of FBP1 in PC9, PC9/GR, HCC827, and HCC827/GR cells. (e). The mRNA and protein levels of FBP1 in PC9/GR and HCC827/GR cells with or without 10 μg/ml β‐elemene treatment. (f) Representative images of the dose‐dependent increase in FBP1 expression after β‐elemene treatment. ** *p* < 0.01

BATMAN‐TCM was carried out to predict the potential genes involved in the function of β‐elemene. A total of 98 potential genes were found (Figure 1a). The GSE123066 datasheet was used to analyze the differentially expressed genes (DEGs) between parental cells and gefitinib‐resistant cells, and the results showed that 326 genes were identified with a fold change >1.5, *p* < 0.01 (Figure 1b). Five common genes (RNASE1, ANK3, SPARC, OXTR, FBP1) were mapped through the Venn diagram between β‐elemene targets and DEGs of GSE123066 (Figure 1c). The NCBI‐Gene database was used to investigate the potential roles of these genes in lung cancer, and FBP1 was selected for further investigation. The mRNA and protein expression levels of FBP1 were determined by qRT–PCR and Western blotting in PC9/GR and HCC827/GR cells. The results indicated that the decreased expression of FBP1 was observed at both the mRNA and protein levels (Figure 1d). Then, qRT–PCR assays were carried out to determine the function of β‐elemene in the mRNA expression level of FBP1, as well as the protein expression level. Our results showed that 10 μg/ml β‐elemene treatment led to a significant increase in FBP1 not only in PC9/GR and HCC827/GR cells but also in parental PC9 and HCC827 cells (Figure 1e). In addition, β‐elemene treatment accounted for dose‐dependent alterations in the expression of FBP1, with no significant change after over 10 μg/ml (Figure 1f). These data suggested that FBP1 might play an important role in the development of gefitinib resistance in NSCLC.

### Overexpression of FBP1 enhanced the sensitivity of PC9/R and HCC827/GR cells to gefitinib in vitro

**FIGURE 2:**
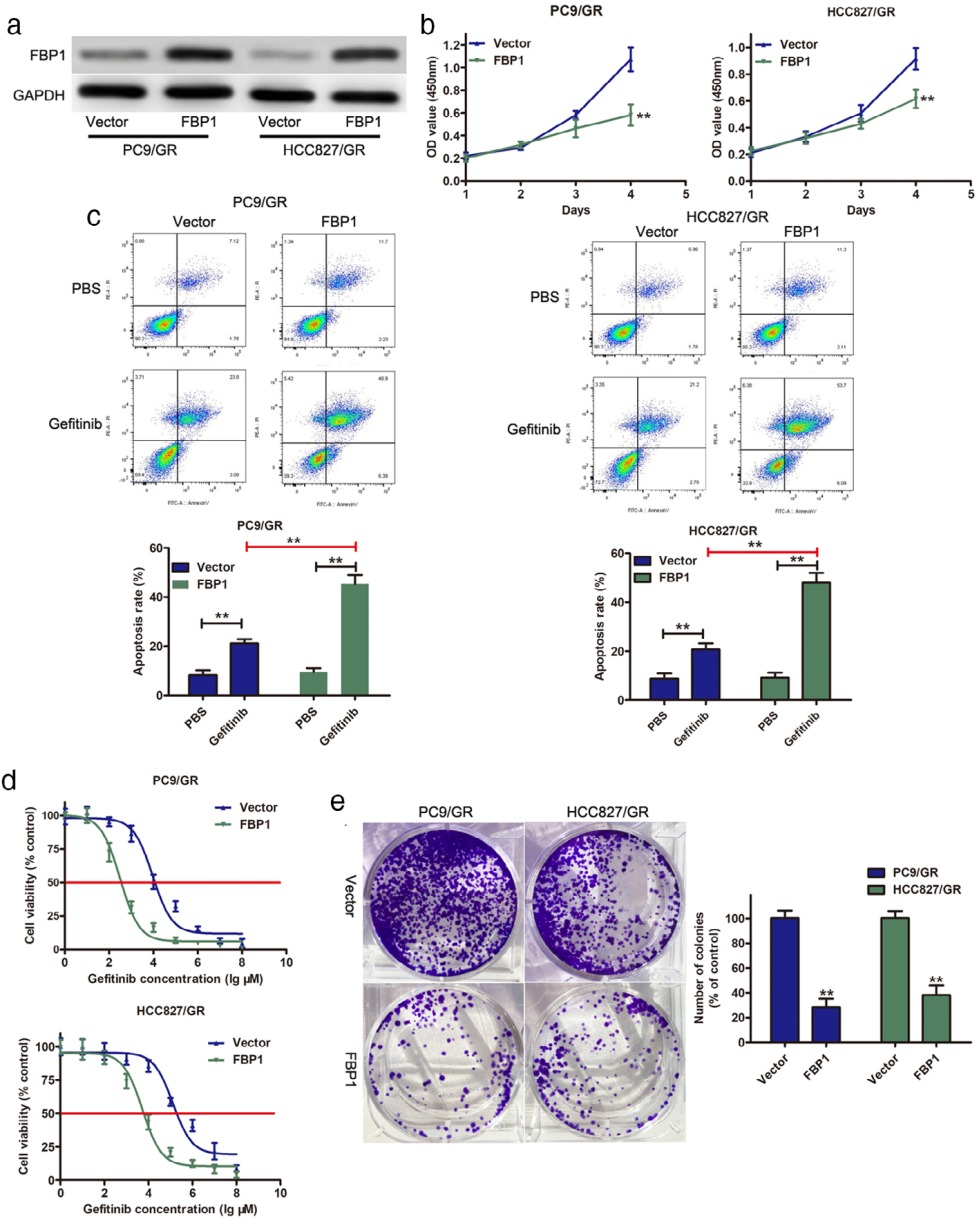
Overexpression of FBP1 inhibited cell growth and induced apoptosis in gefitinib‐resistant lung cells. (a) The expression of FBP1 was examined in PC9/GR and HCC827/GR cells after FBP1 overexpression by Western blot. (b) Growth curve of PC9/GR and HCC827/GR cells after FBP1 overexpression by CCK‐8 assay. (c) Flow cytometry was performed to assess the apoptosis level of PC9/GR and HCC827/GR cells after FBP1 overexpression and 10 μM gefitinib treatment. (d) The IC50 values of gefitinib in PC9/GR and HCC827/GR cells were analyzed using GraphPad. (e) A colony forming experiment was conducted in PC9/GR and HCC827/GR cells after FBP1 overexpression. ** *p* < 0.01

To investigate whether FBP1 was involved in gefitinib resistance, FBP1 was overexpressed in gefitinib‐resistant cell lines (PC9/GR and HCC827/GR). As shown in Figure 2a, FBP1 expression was greatly enhanced in PC9/GR and HCC827/GR cells after transfection with FBP1. CCK‐8 analysis suggested that overexpression of FBP1 led to a significant decrease in cell proliferation (Figure 2b). Flow cytometry assays showed that overexpression of FBP1 could induce cell apoptosis in both PC9/GR and HCC827/GR cells (Figure 2c). Overexpression of FBP1 enhanced gefitinib sensitivity in PC9/GR and HCC827/GR cells, and the IC50 value of gefitinib was significantly decreased in PC9/GR and HCC827/GR cells transfected with FBP1 (Figure 2d). Moreover, a colony formation assay revealed that overexpression of FBP1 resulted in a great decrease in colony number in both PC9/GR and HCC827/GR cells (Figure 2e). These results suggested that FBP1 acted as a tumor suppressor gene to inhibit gefitinib‐resistant cells and that overexpression of FBP1 sensitizes gefitinib‐resistant cells to gefitinib.

### β‐elemene inhibits the proliferation of NSCLC cells dependent on FBP1 expression

**FIGURE 3:**
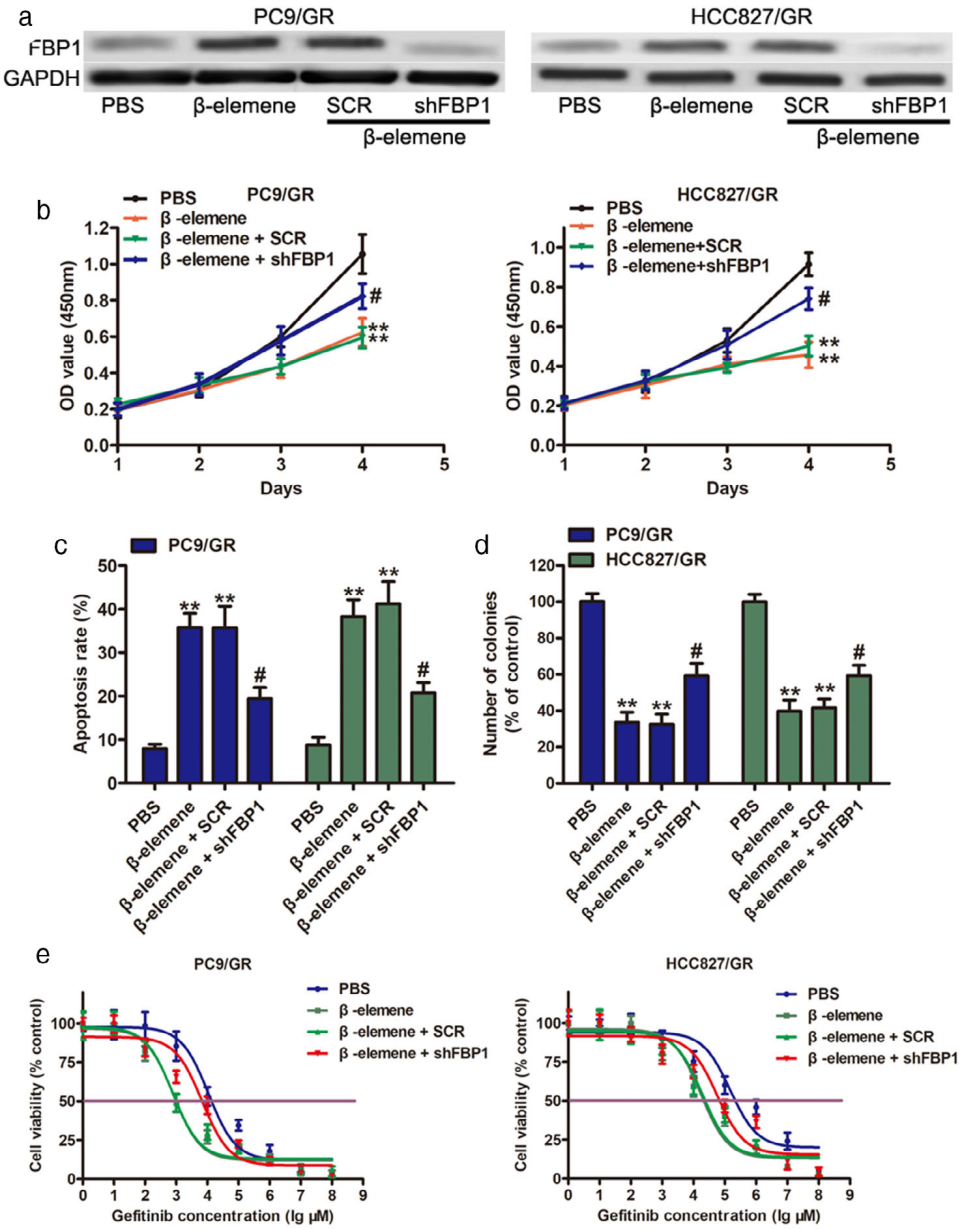
FBP1‐mediated sensitivity to gefitinib in PC9/GR and HCC827/GR cells. PC9/GR and HCC827/GR were transduced with SCR or shFBP1 (shRNA targeting FBP1) and then treated with 10 μg/ml β‐elemene for 24 h for subsequent experiments. (a) The protein level of FBP1 was measured in PC9/GR and HCC827/GR cells. (b) Growth curve, (c) apoptosis level, (d) colony formation, and (e) IC50 values of gefitinib in PC9/GR and HCC827/GR cells as indicated treatment. **p < 0.01, compared with PBS; # *p* < 0.05, compared with SCR

To further investigate the effects of FBP1 required for gefitinib‐resistant PC9/GR and HCC827/GR cells to β‐elemene, shRNAs targeting FBP1 were transfected into PC9/GR and HCC827/GR cells to downregulate FBP1 expression. Western blot analysis indicated that the expression levels of FBP1 were significantly decreased in FBP1‐silenced PC9/GR and HCC827/GR cells, even after β‐elemene treatment (Figure 3a). Subsequent functional assays suggested that FBP1 knockdown enhanced the proliferation of PC9/GR and HCC827/GR cells in the presence of 10 μg/mL β‐elemene (Figure 3b). Furthermore, the results of the flow cytometry assay showed that depletion of FBP1 reduced apoptosis (Figure 3c) and enforced colony forming ability (Figure 3d) in PC9/GR and HCC827/GR cells upon β‐elemene treatment. Moreover, higher IC50 values for gefitinib were observed in FBP1‐silenced PC9/GR and HCC827/GR cells upon β‐elemene treatment. Taken together, FBP1 was induced by β‐elemene and involved in β‐elemene‐mediated chemosensitivity to gefitinib in NSCLC cells.

### STAT3 is involved in FBP1‐mediated cell apoptosis and gefitinib resistance in gefitinib‐resistant lung cells

**FIGURE 4:**
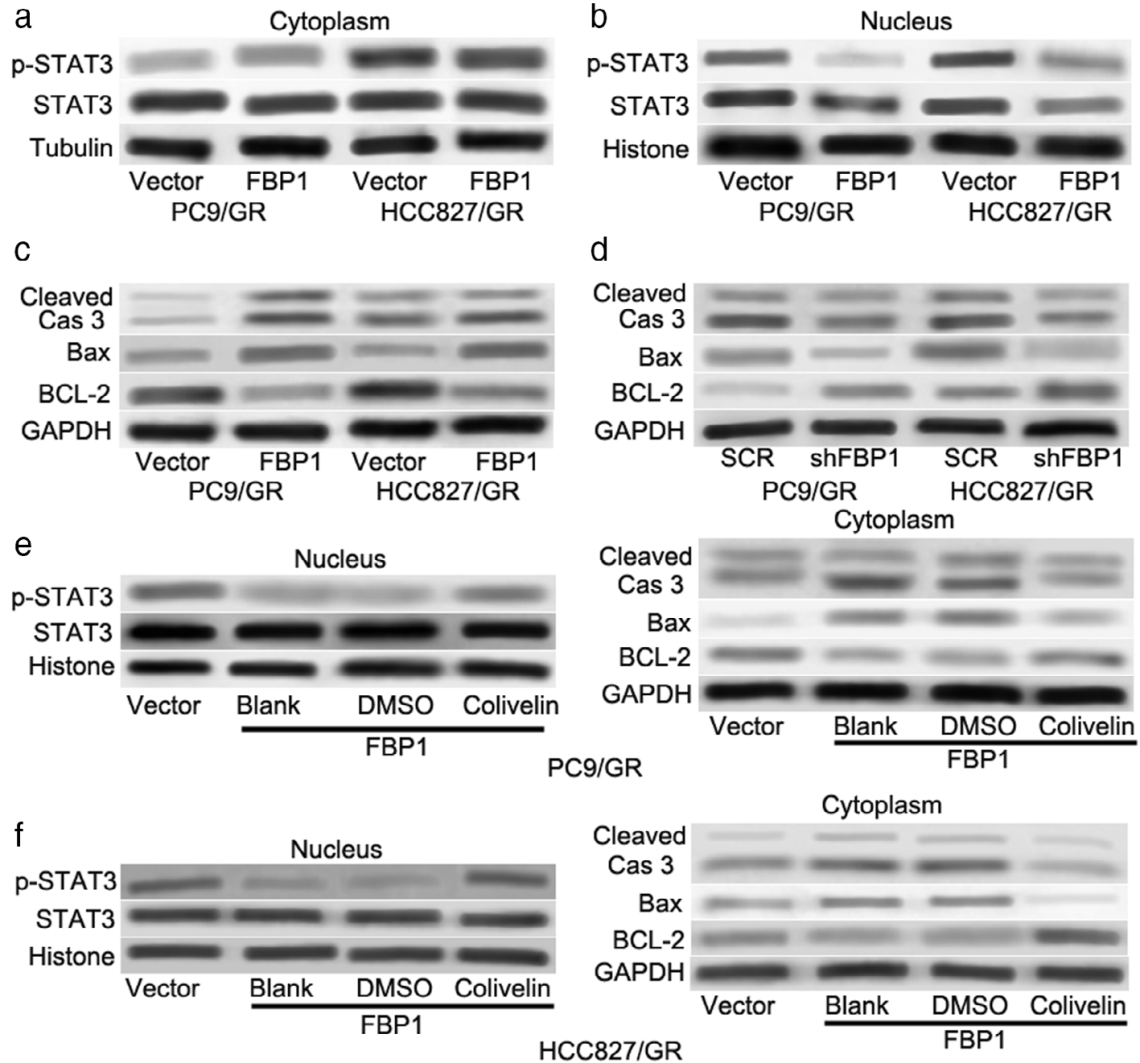
STAT3 was required for the FBP1‐mediated apoptosis pathway. (a) The protein expression of STAT3 and p‐STAT3 (Tyr705) in PC9/GR cells after FBP1 overexpression in the nucleus and cytoplasm. (b) The protein expression of STAT3 and p‐STAT3 (Tyr705) in HCC827/GR cells after FBP1 overexpression in the nucleus and cytoplasm. (c) Western blot analysis was used to evaluate the expression of BCL‐2, Bax, and cleaved caspase‐3 in PC9/GR and HCC827/GR cells after FBP1 overexpression. (d) Western blot analysis was used to evaluate the expression of BCL‐2, Bax, and cleaved caspase‐3 in PC9/GR and HCC827/GR cells after FBP1 silencing. (E and F) the protein expression of STAT3 and p‐STAT3 (Tyr705) in PC9/GR (e) and HCC827/GR (f) cells after FBP1 overexpression with or without colivelin treatment in the nucleus, as well as the expression of BCL‐2, Bax, and cleaved caspase‐3 in the cytoplasm

A previous study demonstrated that FBP1 suppressed the nuclear translocation of signal transducer and activator of transcription 3 (STAT3) through physical interactions with STAT3 and exerted its nonmetabolic enzymatic activity to induce the dysfunction of STAT3 to sensitize cancer cells to cisplatin‐induced apoptosis.^26^ Blocking STAT3 was reported to be a new potential therapeutic strategy for gefitinib resistance in lung cancer.^27^, ^28^ The expression of p‐STAT3 (Tyr705) and STAT3 was determined in gefitinib‐resistant cell lines (PC9/GR and HCC827/GR). As shown in Figure 4a, there was no significant change in the cytoplasm. Interestingly, overexpression of FBP1 not only decreased the expression level of STAT3 but also the expression level of p‐STAT3 in the nucleus (Figure 4b). Furthermore, overexpression of FBP1 dramatically enhanced the protein levels of Bax and cleaved Caspase‐3 but suppressed BCL‐2 protein expression (Figure 4c). On the other hand, knockdown of FBP1 had the opposite effects (Figure 4d). To clarify whether STAT3 was involved in FBP1‐mediated cell apoptosis and gefitinib resistance, colivelin (a potent activator of STAT3) was used to activate STAT3. As shown in Figure 4e,f 1 μM colivelin effectively increased the nuclear expression level of p‐STAT3 but not STAT3. In addition, the protein levels of Bax and cleaved Caspase‐3 induced by FBP1 were dramatically decreased after colivelin treatment (Figure 4e right and Figure 4f right). Colivelin also abrogated the downregulation of BCL‐2 induced by FBP1 overexpression (Figure 4e right and Figure 4f right). These results indicated that STAT3 was required for FBP1‐mediated cell apoptosis and gefitinib resistance in gefitinib‐resistant cells.

### Inactivation of STAT3 by β‐elemene was involved in β‐elemene‐mediated cell apoptosis and gefitinib resistance in gefitinib‐resistant cells

**FIGURE 5:**
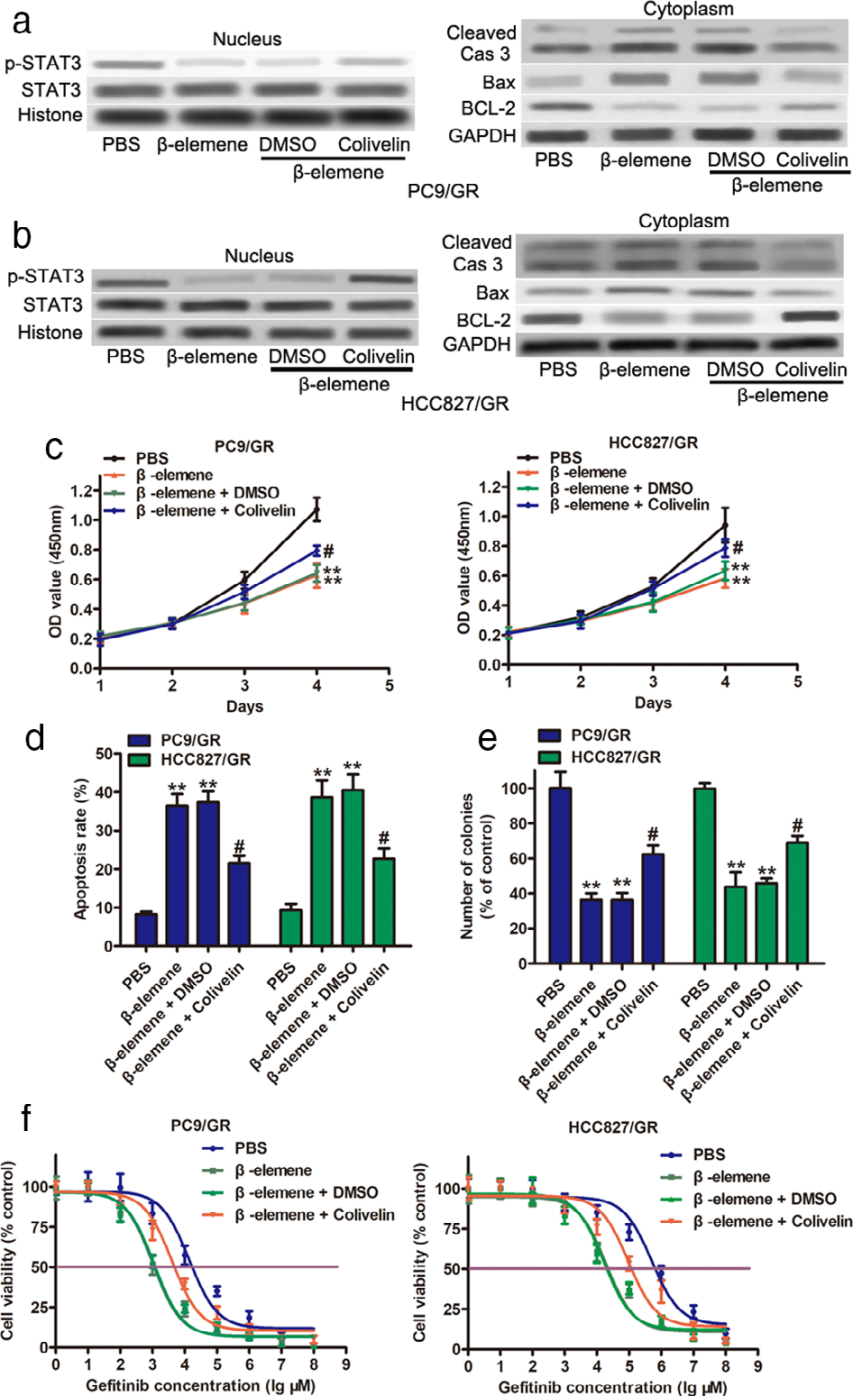
β‐elemene regulates gefitinib resistance through the FBP1/STAT3 axis in PC9/GR and HCC827/GR cells. (a and b) The protein expression of STAT3 and p‐STAT3 (Tyr705) in PC9/GR (a) and HCC827/GR (b) cells after β‐elemene treatment, followed by or without 0.5 μM colivelin treatment in the nucleus. The protein expression of BCL‐2, Bax, and cleaved caspase‐3 in PC9/GR (a) and HCC827/GR (b) cells after β‐elemene treatment, followed by or without colivelin treatment in the cytoplasm. (c) Growth curve, (d) apoptosis level, (e) colony formation, and (f) IC50 values of gefitinib in PC9/GR and HCC827/GR cells as indicated treatment. **p < 0.01, compared with PBS; # *p* < 0.05, compared with DMSO

To investigate whether β‐elemene‐mediated STAT3 was involved in cell apoptosis and gefitinib resistance in gefitinib‐resistant cells, we measured the expression levels of p‐STAT3 and STAT3 in the nucleus of gefitinib‐resistant cells. As shown in Figure 5a,b, the expression level of p‐STAT3 was markedly decreased after β‐elemene treatment but had no impact on the levels of STAT3. Notably, 0.5 μM colivelin partially reversed the inhibition of p‐STAT3 induced by β‐elemene. Western blot assays also showed that β‐elemene treatment enhanced the protein levels of Bax and cleaved Caspase‐3 but suppressed BCL‐2 protein expression (Figure 5a right and Figure 5b right). However, colivelin abolished the alteration of Bax, cleaved Caspase‐3, and BCL‐2 induced by β‐elemene. In addition, functional assays indicated that colivelin attenuated the inhibition of cell proliferation by β‐elemene (Figure 5c). The percentage of apoptotic cells was decreased after colivelin administration (Figure 5d). Moreover, the colony formation assay showed that proliferation in the presence of colivelin was significantly upregulated (Figure 5e). An increased IC50 was observed in PC9/GR and HCC827/GR cells in the presence of β‐elemene that were treated with colivelin (Figure 5f). In summary, our observations suggest that β‐elemene triggers apoptosis and gefitinib sensitivity in gefitinib‐resistant NSCLC cells by suppressing p‐STAT3 activity.

### FBP1 inhibited tumor growth in vivo

**FIGURE 6:**
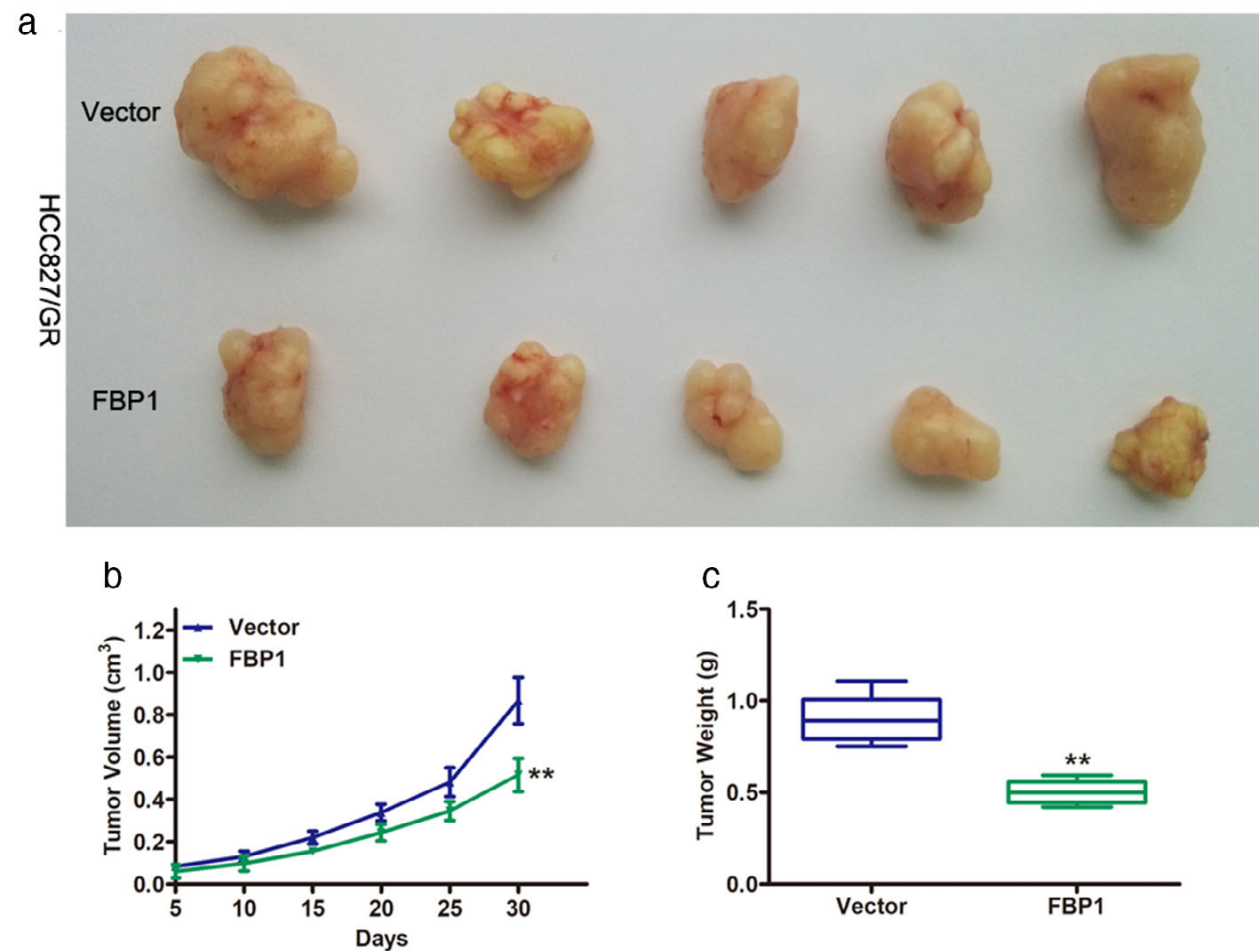
The overexpression of FBP1 inhibited tumor growth in vivo. (a) The xenograft tumors from nude mice with HCC827/GR transduced with vector or FBP1. (b) The growth curve of nude mice in the vector group and FBP1 group. (c) Tumor weight of the xenograft tumors from nude mice with HCC827/GR cells transduced with vector or FBP1. **p < 0.01

To further confirm the roles of FBP1 in gefitinib‐resistant NSCLC cells, HCC827/GR cells transduced with vector or FBP1 were injected into the flanks of BALB/c nude mice. Tumors from the FBP1 group showed significantly smaller volumes than those from the vector group (Figure 6a). Tumor growth in the FBP1 group was remarkably slower than that in the vector group (Figure 6b). In addition, lower tumor weights were observed in the FBP1 group than in the vector group. Taken together, these data further confirmed the repressive role of FBP1 in gefitinib‐resistant NSCLC cells in vitro.

## DISCUSSION

With continuous development, therapeutic strategies, including surgical resection, radiotherapy, chemotherapy and gene‐targeted therapy, for NSCLC have been greatly improved. However, the overall 5‐year survival rate is still low.^29^, ^30^, ^31^ Mutations in EGFR are frequently observed in NSCLC and have become an attractive target in NSCLC treatment.^29^, ^32^ Gefitinib, a first‐generation EGFR‐TKI, showed dramatic clinical efficacy initially and significantly prolonged progression‐free survival in patients harboring these EGFR‐activating mutations.^6^, ^29^, ^33^ However, acquired resistance to TKIs accounted for approximately 60% of NSCLC patients and was usually inevitable in almost all patients after TKI treatment for a median of 10–14 months.^34^, ^35^, ^36^ Therefore, understanding the molecular mechanisms underlying gefitinib resistance requires further investigation to identify new therapeutic strategies for NSCLC treatments.

β‐elemene has been shown to be involved in the chemoresistance of diverse tumors by directly targeting tumor cells or enhancing the therapeutic effect of existing clinical drugs without any side effects.^8^, ^14^ However, the detailed mechanisms related to the reversal of multidrug resistance are unclear. In our present study, FBP1 was identified by bioinformatic analysis, and there was a significant decrease in FBP1 expression in gefitinib‐resistant PC9/GR and HCC827/GR cells compared with parental cells. Gain‐ and loss‐of‐function experiments revealed that overexpression of FBP1 inhibited viability and colony forming capability and enhanced apoptosis in gefitinib‐resistant PC9/GR and HCC827/GR cells, while knockdown of FBP1 had the opposite effects, suggesting that FBP1 participated in gefitinib resistance in NSCLC cells. Consistent with previous results, overexpression of FBP1 increased triple‐negative breast cancer (TNBC) cell sensitivity to paclitaxel treatment.^37^ Moreover, FBP1 was reported to suppress proliferation and invasion, reduce aerobic glycolysis, and enhance epithelial ovarian carcinoma cells to undergo gefitinib‐induced apoptosis in vitro and in vivo.^26^ Inhibition of FBP1 abolished the effects of β‐elemene on gefitinib‐resistant cells, which suggested that FBP1 was required for β‐elemene function.

STAT3, a member of the STAT family, regulates various biological processes, including cell proliferation, metastasis, angiogenesis, chemoresistance, and immune response, through its SH2 domain.^28^, ^38^ A recent study showed that FBP1 could directly bind to the transactivation domain and the domain containing the nuclear translocation signal of STAT3 to retain STAT3 in the cytoplasm.^26^ Our results demonstrated that β‐elemene could reduce the expression level of p‐STAT3 in the nucleus via FBP1, which was consistent with previous studies.^39^, ^40^ Subsequent experiments showed that activation of STAT3 could attenuate the effects of β‐elemene on gefitinib‐resistant cells, which indicated that STAT3 was involved in β‐elemene function. Anti‐apoptosis is a feature of chemoresistance, and β‐elemene exerts anti‐proliferative and anti‐apoptosis effects on tumor growth and multidrug resistance.^9^, ^14^ Previous studies have shown that BCL‐2 is transcriptionally regulated by STAT3.^41^, ^42^ In our present manuscript, colivelin, an activator of STAT3, effectively attenuated the inhibition of BCL‐2 protein expression induced by β‐elemene, which indicated that the FBP1/stat3 pathway was crucial for the functions of β‐elemene.

**FIGURE 7:**
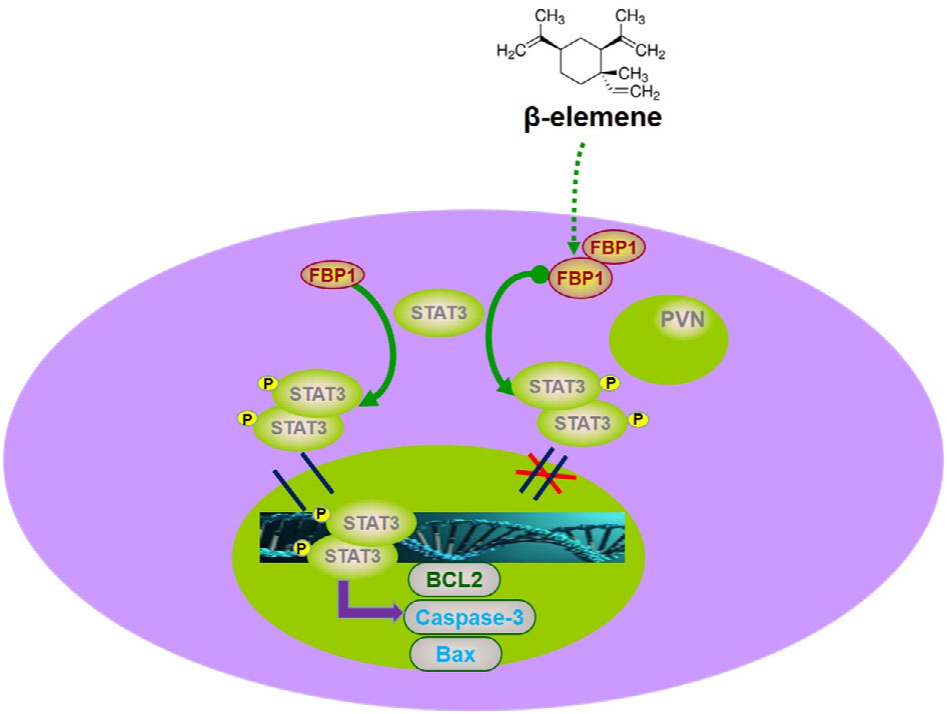
The working model describes the mechanism of FBP1. The expression of FBP1 was increased in gefitinib‐resistant lung cancer cells when stimulated by β‐elemene. The upregulation of FBP1 bound to STAT3 and inhibited the phosphorylation of STAT3, resulting in the altered expression of apoptosis‐related genes and leading to gefitinib sensitivity

In conclusion, our data provide evidence that FBP1 was induced by β‐elemene and inhibited the proliferation and gefitinib resistance of PC9/GR and HCC827/GR cells by inducing apoptosis. In addition, we have demonstrated a role of the FBP1/STAT3 axis in the functions of β‐elemene in gefitinib‐resistant NSCLC (Figure 7), which may be a promising target for therapeutic intervention in patients with gefitinib resistance.

## AUTHOR CONTRIBUTIONS

KCL: Conception and design, KCL: Administrative support; JL and PD: Cell culture studies, molecular biology experiments, data collection, statistical analysis; JS, YWY and WH: Participated in the molecular biology experiments.

## CONFLICT OF INTEREST

The authors declare no competing interests.

## Data Availability

The RNA‐Seq data reported in this article have been deposited under the gene expression omnibus (GEO) accession number GSE.
